# NOV/CCN3 induces cartilage protection by inhibiting PI3K/AKT/mTOR pathway

**DOI:** 10.1111/jcmm.14621

**Published:** 2019-08-27

**Authors:** Xiaojian Huang, Bowei Ni, Zekai Mao, Yang Xi, Xiangyu Chu, Rui Zhang, Xiaohu Ma, Hongbo You

**Affiliations:** ^1^ Department of Orthopedics, Tongji Hospital, Tongji Medical College Huazhong University of Science and Technology Wuhan China

**Keywords:** CCN3, HMGB1, IL‐1β, MMPs, osteoarthritis

## Abstract

Osteoarthritis (OA), an age‐related degenerative joint disease, is pathologically characterized by articular cartilage degeneration and synovial inflammation. Nephroblastoma overexpressed (NOV or CCN3), a matricellular protein, is a primary member of the CCN family (Cyr61, Ctgf, NOV) of proteins and is involved in various inflammatory disorders. Previous studies reported that CCN3 might play a therapeutic role in OA. However, the underlying mechanism remains unclear. In this study, we confirmed the expression of CCN3 was decreased in human and rat OA articular cartilage. Recombinant CCN3 ameliorated the IL‐1β‐induced matrix catabolism, as demonstrated by MMP1, MMP3, MMP13, ADAMTS5 and iNOS expression, in vitro. In addition, the degradation of cartilage matrix such as collagen 2 and aggrecan could be reversed by CCN3. Furthermore, we found CCN3 promoted autophagy as Atg5, Beclin1 and LC3‐II expression were increased. High‐mobility group box 1 was negatively correlated with CCN3 in IL‐1β‐induced osteoarthritis responses, and HMGB1 is involved in the protective effect of CCN3 in OA. Moreover, CCN3 overexpression decreased the expression of HMGB1 and reversed the IL‐1β induced MMPs production. Additionally, recombinant CCN3 or CCN3 overexpression attenuated the activation of PI3K/AKT/mTOR pathway induced by IL‐1β. Our study presents new mechanisms of CCN3 in osteoarthritis and indicates that CCN3 can serve as a novel potential therapeutic target for osteoarthritis.

## 1 INTRODUCTION

Osteoarthritis (OA) is a common joint disorder involving articular cartilage degeneration and synovial inflammation.^1^ Osteoarthritis is one of the most common causes of pain and disability among ageing populations worldwide.^2^ According to epidemiology studies, half of the world's population over 65 years old suffers from OA.^3^ In recent decades, the incidence and prevalence of OA has skyrocketed significantly because of increased life expectancy.^4^ Although disease initiation may be multi‐factorial, cartilage destruction is thought to be a result of uncontrolled proteolytic extracellular matrix destruction.^5^ Matrix metalloproteinases (MMPs) and a disintegrin and metalloproteinase with thrombospondin motifs (ADAMTS) are zinc‐dependent endopeptidases that mediate the degradation of cartilage extracellular (ECM) proteins.^6^ Thus, strategies limiting the release of MMPs may provide new methods for the treatment of OA.

High‐mobility group box 1 (HMGB1) is a widely‐expressed and highly‐abundant nuclear protein participating in DNA replication, transcription, recombination and repair.^7^ Accumulating evidence indicates that HMGB1 can be passively released from dead/ damaged cells during inflammation and tissue injury.^8^ Cytosolic HMGB1 is a multifunctional cytokine that is involved in the process of inflammation, apoptosis and autophagy.^9^ High‐mobility group box 1 has been documented as a significant contributor to the progression of OA.^10^ Damage to chondrocytes within the cartilage allows the release of HMGB1, and HMGB1 increases the secretion of pro‐inflammatory cytokines and MMPs, leading to cartilage degeneration via RAGE and TLRs pathway.^11^

Nephroblastoma overexpressed (NOV or CCN3), a matricellular protein, is a primary member of the CCN family (Cyr61, Ctgf, NOV) of proteins.^12^ The CCN3 gene was originally identified as an integration site from avian nephroblastomas induced by viral infections.^13^ As a secreted protein, CCN3 has both the characteristics of conventional cytokines and extracellular matrix molecules. CCN3 not only acts through their own putative receptors, but also alter the effects of various growth factors and cytokines.^12^, ^14^ Previous studies have shown that CCN3 is involved in various cellular functions such as cell division, apoptosis, adhesion, proliferation and differentiation.^15^ CCN3 also modulates the expression of inflammatory molecules in astrocyte.^16^ Recently, emerging evidence have indicated that CCN3 may act as a therapeutic target in OA.^17^ Mice lacking CCN3 protein have been shown to display osteoarthritic changes in knee articular cartilage.^18^ CCN3 may inhibit osteoarthritis progression by maintaining the differentiated phenotype of articular cartilage.^19^ However, the exact mechanism by which CCN3 alleviates osteoarthritis remains unknown.

## 2 MATERIALS AND METHODS

### 2.1 Cell culture and reagents

Recombinant CCN3 protein and recombinant human IL‐1β were purchased from R&D systems. SW1353 human chondrosarcoma cell lines were obtained from the Institute of Life Science Cell Culture Centre.

Rat chondrocytes were obtained from the knee joints of 1‐week‐old Sprague Dawley rats by sequential enzymatic digestion, as previously described.^20^ Cells were grown in Dulbecco's modified Eagle's medium (DMEM; Gibco) containing 10% foetal bovine serum and 100 U/mL penicillin and 100 μg/mL streptomycin at 37°C, in a humidified atmosphere containing 5% CO2.

### 2.2 Patients selection and informed consent

Osteoarthritis human articular cartilage specimens were collected from patients (n = 6) with knee OA undergoing total knee arthroplasty. Normal human articular cartilage specimens were collected from patients (n = 6) without knee arthropathy undergoing lower limb amputation because of trauma. X‐ray and Kellgren‐Lawrence Grading Scale were used here to assess the stage of OA. Informed consent forms were signed by all patients. Cartilage tissues were cut into 5‐μm sagittal sections and embedded in paraffin for histological analysis. All experimental procedures were approved by Ethical Committee of Tongji Medical College, Huazhong University of Science and Technology.

### 2.3 Animal model

Twelve 8‐week‐old male Sprague Dawley rats were purchased from the Animal Care and Use Committee of Tongji Medical College. A rat OA model was conducted by surgical destabilization of the anterior cruciate ligament transection (ACLT) and medial meniscus (DMM) of the right knee joint. Rats were divided to a sham group and an ACLT + DMM group. The joint capsule of the sham operation group was opened without transaction of the ACLT and DMM. Twelve weeks after surgery, the rats were killed and the joints were collected for histological evaluation. All the animal procedures were approved and under supervision by the Experimental Animal Ethics Committee of Tongji Medical College, Huazhong University of Science and Technology.

### 2.4 Lentivirus transfection

CCN3 was overexpressed via transfection of lenti‐CCN3 (GeneChem). Cells were seeded in 6‐wells plate and were transfected with lenti‐CCN3 or lenti‐NC at a confluence of 50%. Fourteen hours after incubation, the medium was changed and cells were cultured for further experiments. The transfection efficacy of lenti‐CCN3 was detected by western blotting.

### 2.5 Western blot analysis

Protein concentrations in the supernatants were measured using a BCA protein assay kit (Boster). Then, equal amounts of protein lysate were loaded on a 10% SDS‐polyacrylamide gel and transferred to polyvinylidene fluoride membranes (Millipore). The membranes were blocked with 5% BSA at 37°C for 60 minutes, and incubated with primary antibodies overnight at 4°C. The following primary antibodies were used in this study: MMP1 (#10371‐2‐AP; 1:1500), MMP3 (#17873‐1‐AP; 1:1000), HMGB1 (#10829‐1‐AP; 1:1000), TLR4 (#19811‐1‐AP; 1:1000) and PI3K (#20584‐1‐AP; 1:1000) from Proteintech Group; Collagen2 (#ab34712; 1:1000), MMP13 (#ab39012; 1:1000), iNOS (#ab3523; 1:500), Aggrecan (#ab3778; 1:1000) from Abcam (Cambridge, MA); CCN3 (#8767; 1:1000), P‐AKT (#4060; 1:1000), AKT (#4685; 1:1000), P‐mTOR (#5536; 1:1000), mTOR (#2983; 1:1000), P‐PI3K (#4228; 1:1000), Atg5 (#12994; 1:1000), Beclin1 (#3495; 1:1000) and LC3‐I/II (#12741; 1:1000) from Cell Signalling Technology; ADAMTS5 (#BA3020; 1:500) and GAPDH (#BM3876; 1:500) from Boster. Next, the blots were incubated with goat anti‐rabbit or goat anti‐mouse IgG secondary antibodies (Boster, 1;5000) for 1 hour at 37°C. Protein bands were then detected using a super ECL chemiluminescence solution (Boster). The protein expression was normalized to GAPDH and quantified using ImageJ software.

### 2.6 Histochemistry and immunohistochemistry

Human and rat articular cartilages sections were stained with hematoxylin and eosin (H&E) as well as Safranin‐O‐Fast Green. The paraffin‐embedded tissue sections were firstly deparaffinized and blocked with 5% BSA. Next, primary antibodies against CCN3 (1:100, 55449‐1‐AP, Proeteintech) and HMGB1 (1:200, 10829‐1‐AP, Proteintech) were applied and incubated overnight at 4°C. Finally, slides were incubated with conjugated secondary antibodies for 1 hour at 37°C. Images of the histology slides were captured under a light microscope, and the number of CCN3 positive cells was quantified.

### 2.7 Immunofluorescence

The treated cells were fixed with 4% paraformaldehyde for 15 minutes, permeabilized with 0.5% Triton X‐100 for 10 minutes and blocked with 1% BSA for 1 hour at 37°C. Primary antibodies against HMGB1 (1:100, 10829‐1‐AP, Proteintech) were applied and incubated overnight at 4°C. Next, cells were incubated with CY3‐goat anti‐rabbit IgG (1:100, BA1032, Boster) for 1 hour in dark, and diamidino‐2‐phenylindole (DAPI) was added for 10 minutes to stain the nuclei. Protein expression was quantified by integrated optical density (IOD) with the Image‐J image analysis system.

### 2.8 Statistical analysis

All data are displayed as means ± standard deviation (SD). Student's *t* test or one‐way analysis of variance (ANOVA) were utilized to determine the statistical significance among different groups. *P* ≤ .05 was deemed statistically significant.

## 3 RESULTS

### 3.1 CCN3 expression is decreased in human and rat knee OA articular cartilage

**Figure 1:**
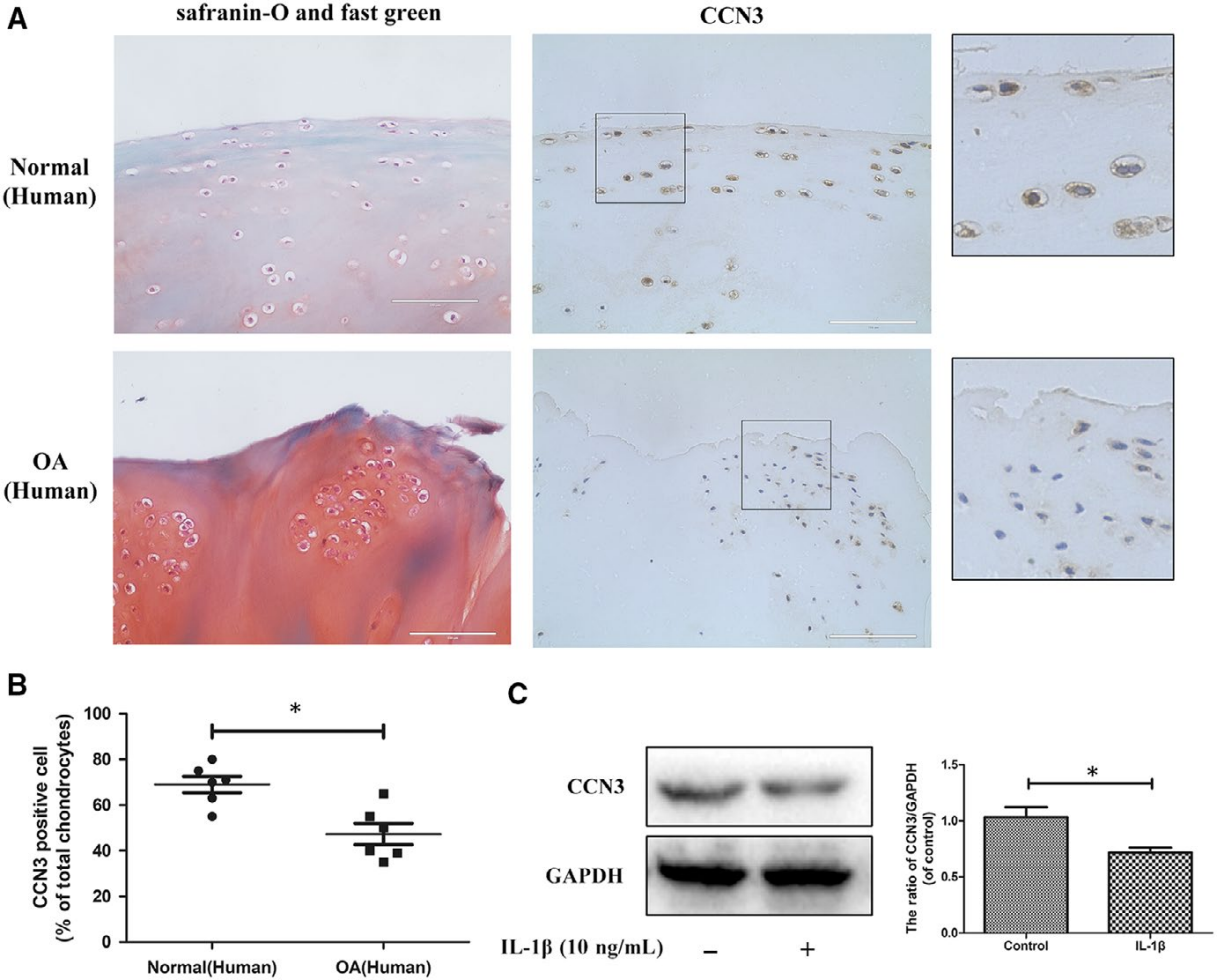
CCN3 expression is decreased in human knee OA articular cartilage. A, Safranin‐O and fast green staining showed significant cartilage erosion in human OA cartilage compared with normal cartilage. Immunohistochemistry showed decreased CCN3 expression in human OA cartilage. B, Quantitative analysis showed lower CCN3 expression in OA cartilage than in healthy cartilage (n = 6). C, Western blot and quantitative analysis showed lower CCN3 expression in SW1353 cells stimulated with IL‐1β for 24 h than in normal cells. (original magnification × 40, scale bar: 100 μm). Significant differences between groups are indicated as ***P < .001, **P < .01 and *P < .05

**Figure 2:**
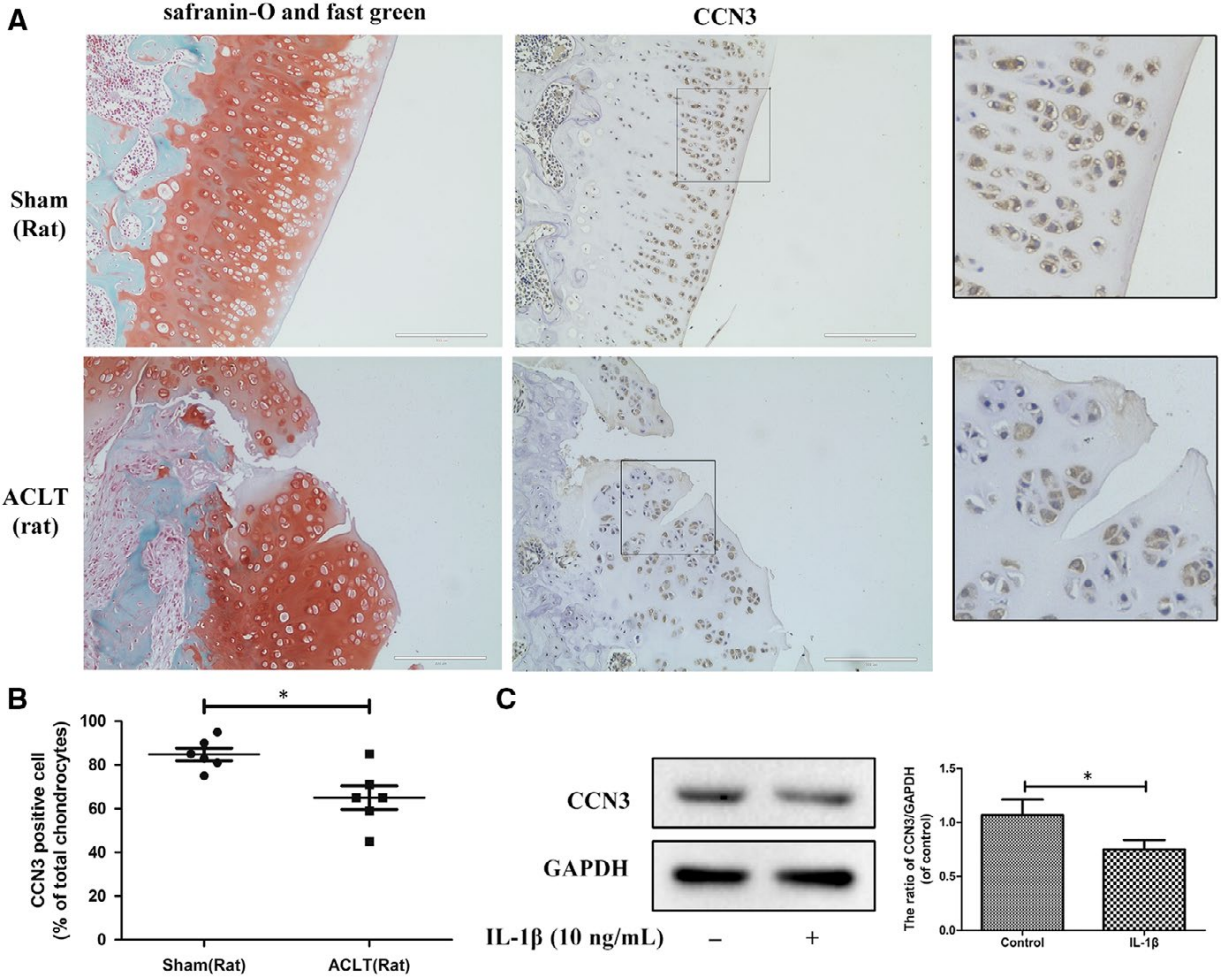
CCN3 expression is decreased in rat knee OA articular cartilage. A, Safranin‐O and fast green staining showed significant cartilage erosion in rat OA cartilage compared with normal cartilage. Immunohistochemistry showed decreased CCN3 expression in rat OA cartilage. B, Quantitative analysis showed lower CCN3 expression in OA cartilage than in normal cartilage (n = 6). C, Western blot and quantitative analysis showed lower CCN3 expression in rat chondrocytes stimulated with IL‐1β for 24 h than in normal cells. (original magnification × 40, scale bar: 100 μm). Data represent mean ± SD of three independent experiments, each done in triplicate. Significant differences between groups are indicated as ***P < .001, **P < .01 and *P < .05

To determine changes in CCN3 levels in OA, western blot and immunohistochemistry were applied to human and rat knee OA articular cartilage tissue and chondrocytes. In Figure 1A, Safranin‐O‐Fast Green staining showed significant cartilage erosion in OA cartilage. According to immunohistochemistry results, a decrease in CCN3 expression was observed in human OA cartilage. After SW1353 cells were treated with IL‐1β for 24 hours, the CCN3 expression was decreased according to western blot analysis (Figure 1C). Similar results were observed in rat cartilage chondrocytes as well as in the sham and rat OA cartilage tissues (Figure 2A‐C). We observed decreased expression of CCN3 in rat OA cartilage compared with that in sham group. CCN3 expression was decreased after treatment with IL‐1β for 24 hours in rat chondrocytes.

### 3.2 CCN3 ameliorates the IL‐1β‐induced catabolism, cartilage matrix degradation and promotes autophagy process in vitro

**Figure 3:**
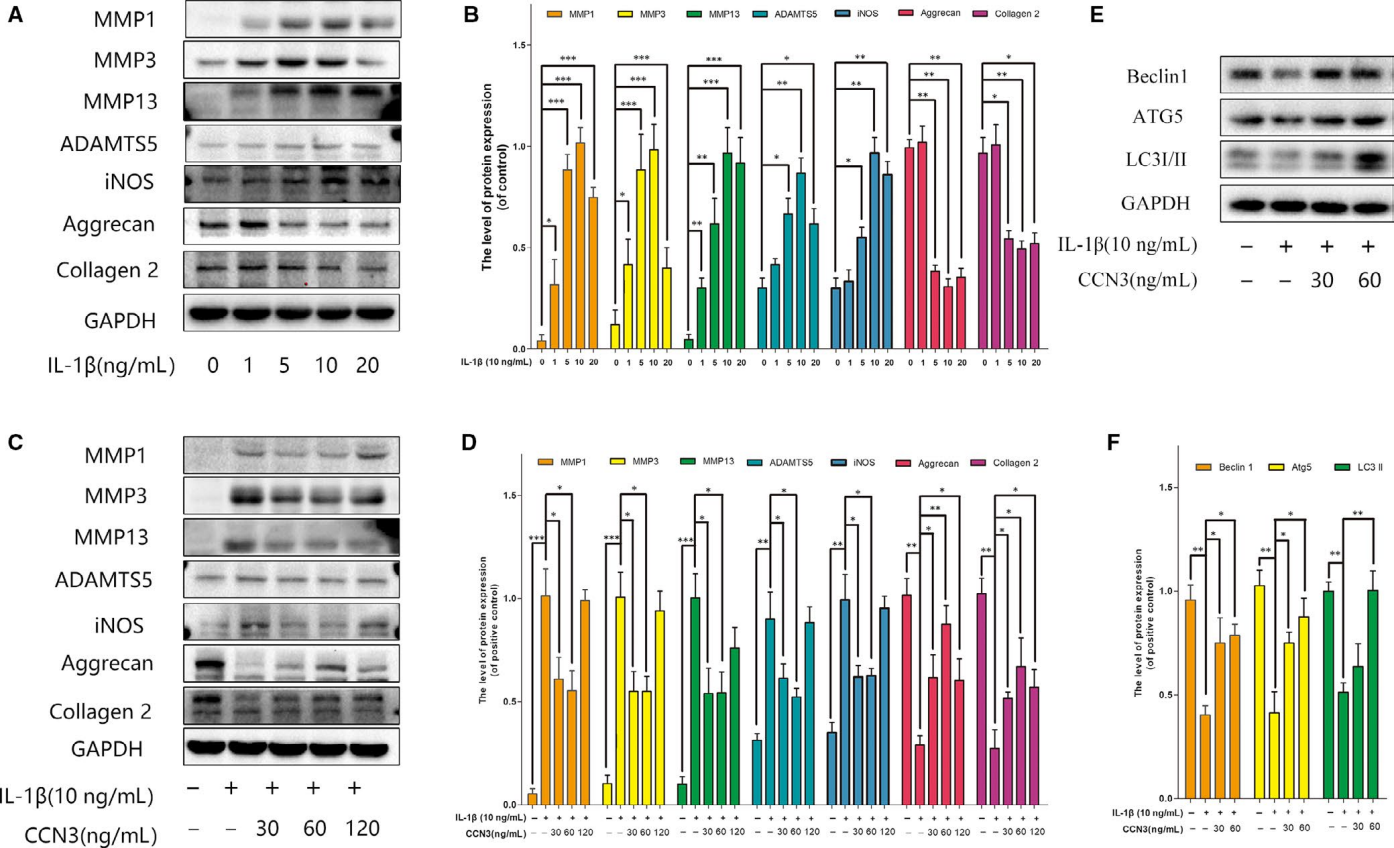
CCN3 ameliorates the IL‐1β‐Induced catabolism, cartilage matrix degradation and promotes autophagy process in vitro. A, SW1353 cells were treated by IL‐1β at different doses for 24 h. Western blotting was performed to examine changes in MMPs, ADAMTS5, iNOS, collagen 2 and aggrecan expression. B, Quantification of MMP, ADAMTS5, iNOS, collagen 2 and aggrecan immunoblots. C, SW1353 cells were treated with recombinant CCN3 (30, 60 and 120 ng/mL) and IL‐1β (10 ng/mL) for 24 h. Western blotting was performed to examine changes in MMPs, ADAMTS5, iNOS, collagen 2 and aggrecan expression. D, Quantification of MMP, ADAMTS5, iNOS, collagen 2 and aggrecan immunoblots. E, SW1353 cells were treated with recombinant CCN3 (30, 60 ng/mL) and IL‐1β (10 ng/mL) for 24 h. Western blotting was performed to examine changes in Atg5, Beclin1 and LC3‐I/II expression. F, Quantification of Atg5, Beclin1 and LC3‐I/II immunoblots. Data represent mean ± SD of three independent experiments, each done in triplicate. Significant differences between groups are indicated as ***P < .001, **P < .01 and *P < .05

The overproduction of catabolic factors such as MMPs, ADAMTS5, iNOS lead to the degradation of extracellular matrix. During OA, autophagy is also increased to regulate changes in OA‐like gene expression. To determine the role of CCN3 in OA, SW1353 cell were treated with recombinant CCN3 (30, 60, 120 ng/mL) and IL‐1β for 24 hours. As shown in Figure 3A,B, western blot analysis indicated that IL‐1β increased MMP1, MMP3, MMP13, ADAMTS5, iNOS accumulation and decreased collagen 2 and aggrecan expression in a dose‐dependent manner, and 10 ng/mL IL‐1β was the optimal concentration to simulate the OA‐like changes in vitro. However, recombinant CCN3 (30, 60 ng/mL) reversed induced IL‐1β changes in MMPs, ADAMTS5, iNOS, collagen 2 and aggrecan expression (Figure 3C,D). We also observed that recombinant CCN3 could reverse the decreased expression of autophagy markers such as Atg5, Beclin1 and LC3‐II induced by IL‐1β (Figure 3E,F).

### 3.3 HMGB1 is negatively correlated with CCN3 in IL‐1β induced osteoarthritis responses

**Figure 4:**
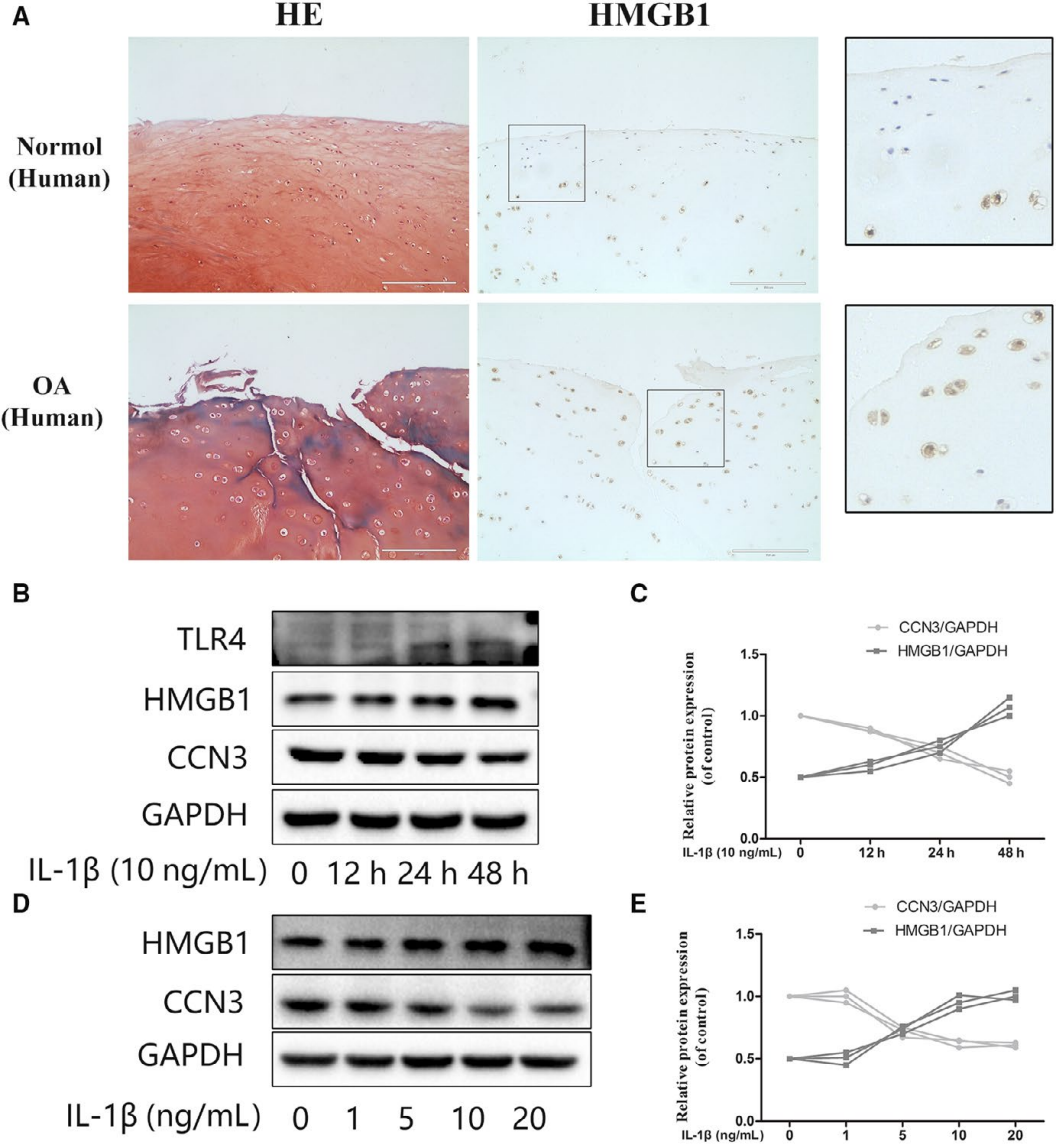
HMGB1 is negatively correlated with CCN3 in IL‐1β induced osteoarthritis responses. A, HE staining showed significant cartilage erosion in human OA cartilage compared with normal cartilage. Immunohistochemistry showed higher HMGB1 expression in human OA cartilage than in normal cartilage. (original magnification × 40, scale bar: 100 μm) B, SW1353 cells were treated with IL‐1β (10 ng/mL) at different time points. Western blot was performed to examine changes in TLR4, HMGB1 and CCN3 expression. C, Quantitative analysis showed HMGB1 is negatively correlated with CCN3 in IL‐1β treated SW1353 cells in a time‐dependent manner. D, SW1353 cells were treated with IL‐1β at different dose points for 24 h. Western blot was performed to examine changes in HMGB1 and CCN3 expression. E, Quantitative analysis showed HMGB1 is negatively correlated with CCN3 in IL‐1β‐induced osteoarthritis responses in a dose‐dependent manner. The data represent mean ± SD of three independent experiments, each done in triplicate. Significant differences between groups are indicated as ***P < .001, **P < .01 and *P < .05

Previous studies have demonstrated that HMGB1 proteins are secreted abundantly in OA. High‐mobility group box 1 is not only an important inflammatory mediator of OA but also a potent regulator of autophagy in chondrocytes. Thus, we have been suggested that CCN3 is associated with HMGB1 levels. As shown in Figure 4A, we observed the significant increased HMGB1 expression in human OA cartilage according to immunohistochemistry results. As shown in Figure 4B‐E, western blot analysis revealed that HMGB1 is negatively correlated with CCN3 in IL‐1β‐treated SW1353 cells in both time‐ and dose‐dependent manners. TLR4, one of the main HMGB1 receptors, was also changed with increased HMGB1 levels.

### 3.4 HMGB1 is involved in the protective effect of CCN3 in osteoarthritis

**Figure 5:**
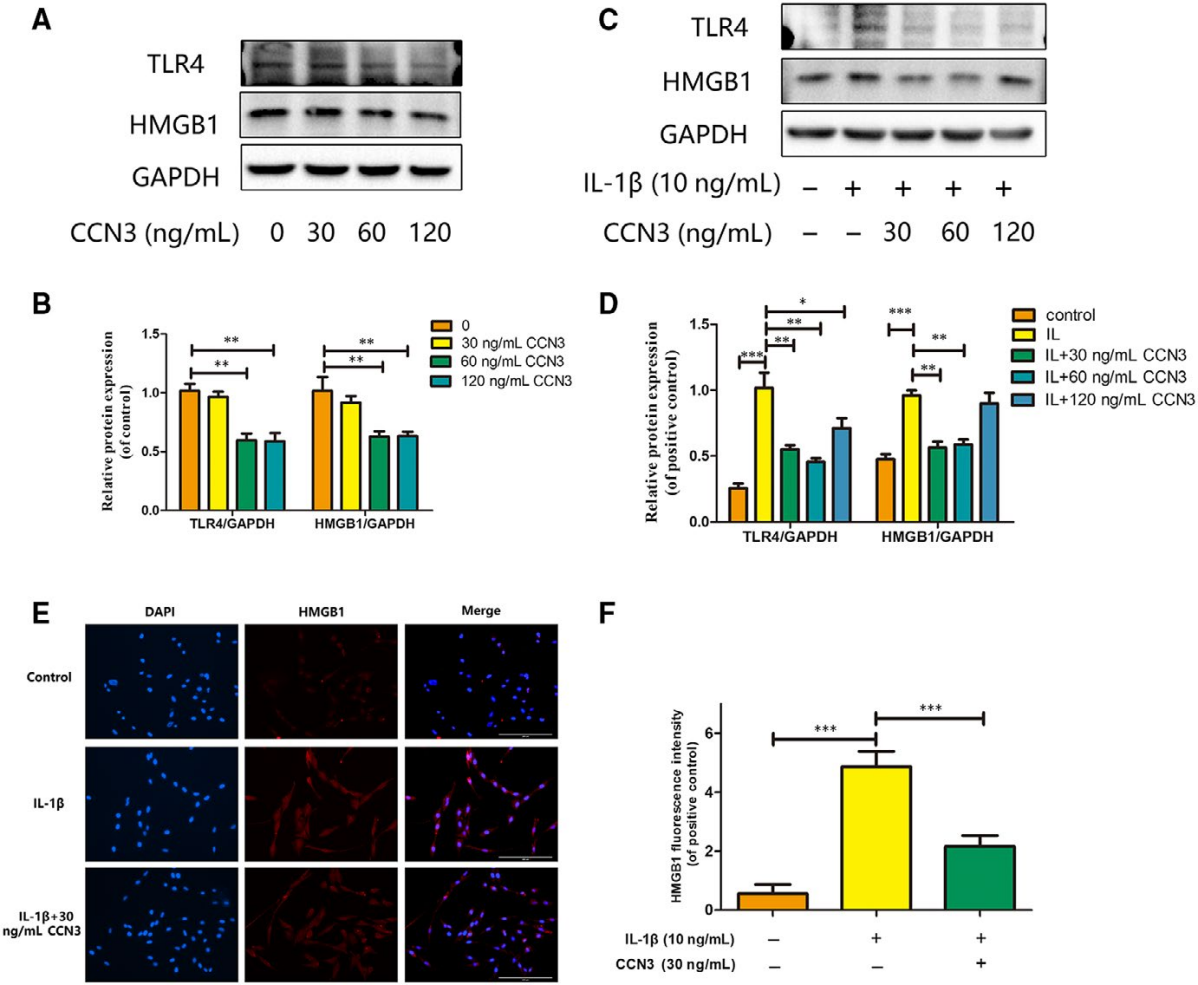
HMGB1 is involved in the protective effect of CCN3 in osteoarthritis. A, SW1353 cells were treated by recombinant CCN3 (30, 60 and 120 ng/mL) for 24 h. Western blot was performed to examine changes in TLR4 and HMGB1 expression. B, Quantitative analysis showed that recombinant CCN3 dose‐dependent decreased HMGB1 and TLR4 expression. C, SW1353 cells were treated with recombinant CCN3 (30, 60 and 120 ng/mL) and IL‐1β (10 ng/mL) for 24 h. Western blot was performed to examine changes in HMGB1 and CCN3 expression. D, Quantitative analysis showed recombinant CCN3 dose‐dependent decreased the expression of HMGB1 as well as TLR4. D, Quantitative analysis showed CCN3 (30, 60 ng/mL) reversed the increased HMGB1 and TLR4 production induced by IL‐1β. E, SW1353 cells were treated with 30 ng/mL recombinant CCN3 and IL‐1β (10 ng/mL) for 24 h. Immunofluorescence showed CCN3 reduced the amount of HMGB1. F, The fluorescence intensity of HMGB1 was quantified. Data represent mean ± SD of three independent experiments, each done in triplicate. Significant differences between groups are indicated as ***P < .001, **P < .01 and *P < .05

To determine the relationship between HMGB1 and CCN3 in osteoarthritis, western blot and immunofluorescence assays were used. As shown in Figure 5A,B, recombinant CCN3 dose‐dependent decreased HMGB1 and TLR4 expression. CCN3 (30, 60 ng/mL) reversed the increased HMGB1 and TLR4 production induced by IL‐1β (Figure 5C,D). Moreover, as shown in Figure 5E,F, CCN3 reduced the amount of HMGB1.

### 3.5 CCN3 overexpression decreases the expression of HMGB1 and reverses the IL‐1β‐induced MMP expression

**Figure 6:**
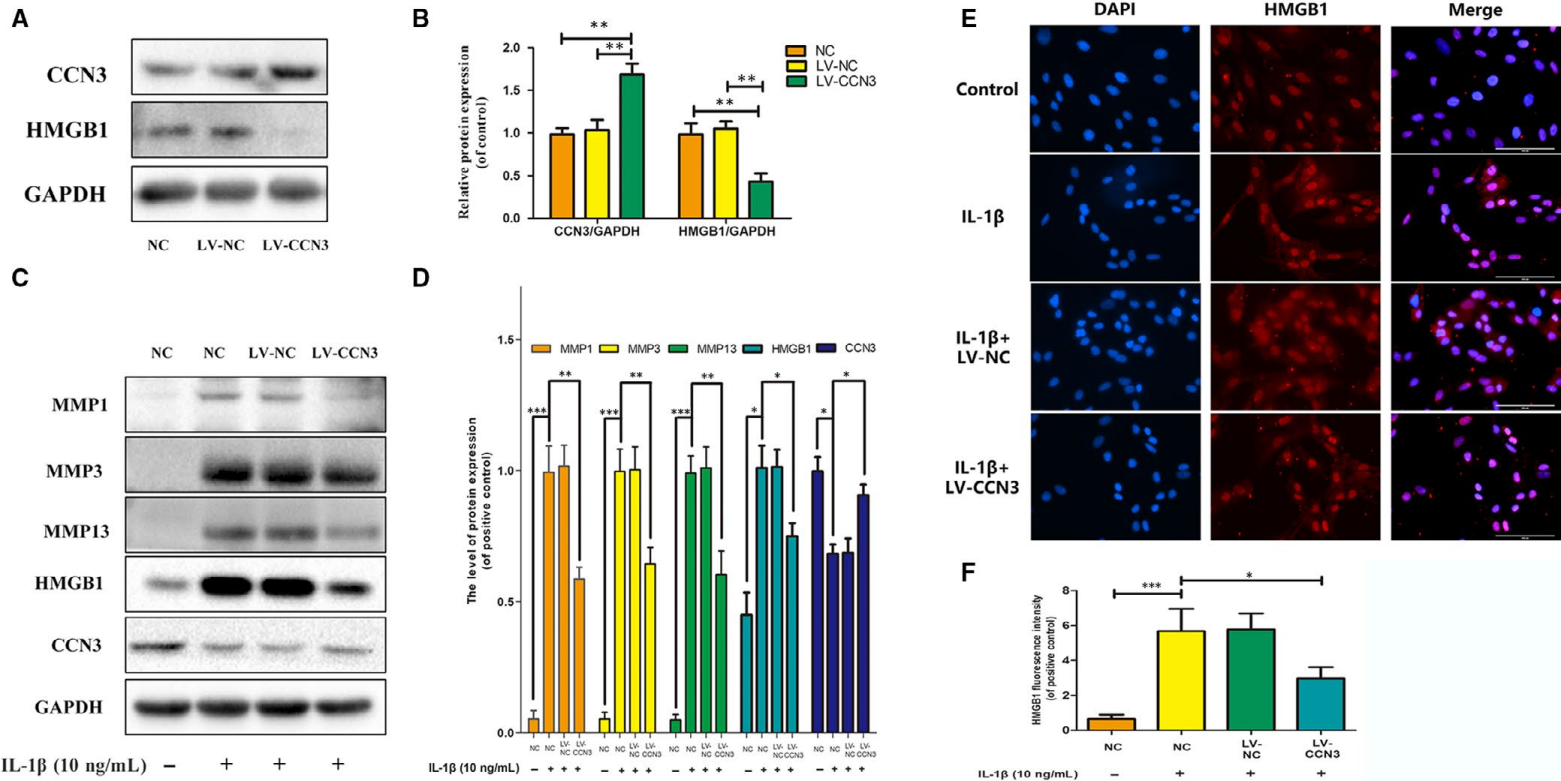
CCN3 overexpression decreases the expression of HMGB1 and reverses the IL‐1β induced MMPs. A, CCN3 was overexpressed via transfection of lenti‐CCN3. Western blot was performed to examine changes in CCN3 and HMGB1 expression. B, Quantitative analysis showed CCN3 overexpression could decrease the HMGB1 expression. C, CCN3 overexpression cells were treated with IL‐1β (10 ng/mL) for 24 h. Western blot showed CCN3 overexpression attenuated the increased MMP1, MMP3, MMP13 and HMGB1 expression induced by IL‐1β. D, Quantification of MMP1, MMP3, MMP13, HMGB1 and CCN3 immunoblots E, Immunofluorescence showed CCN3 overexpression reduced the amount of HMGB1. F, The fluorescence intensity of HMGB1 was quantified. Data represent mean ± SD of three independent experiments, each performed in triplicate. Significant differences between groups are indicated as ***P < .001, **P < .01 and *P < .05

To further investigate the relationship of CCN3 and HMGB1, CCN3 was overexpressed via transfection of lenti‐CCN3. As shown in Figure 6A,B, overexpression of CCN3 could decrease the protein levels of HMGB1. Furthermore, CCN3 overexpression attenuated the increased MMP1, MMP3, MMP13 and HMGB1 expression induced by IL‐1β (Figure 6C,D). Moreover, HMGB1 release induced by IL‐1β could also be inhibited by CCN3 overexpression (Figure 6E,F).

### 3.6 CCN3 induces cartilage protection via blocking the PI3K/AKT/mTOR pathway

**Figure 7:**
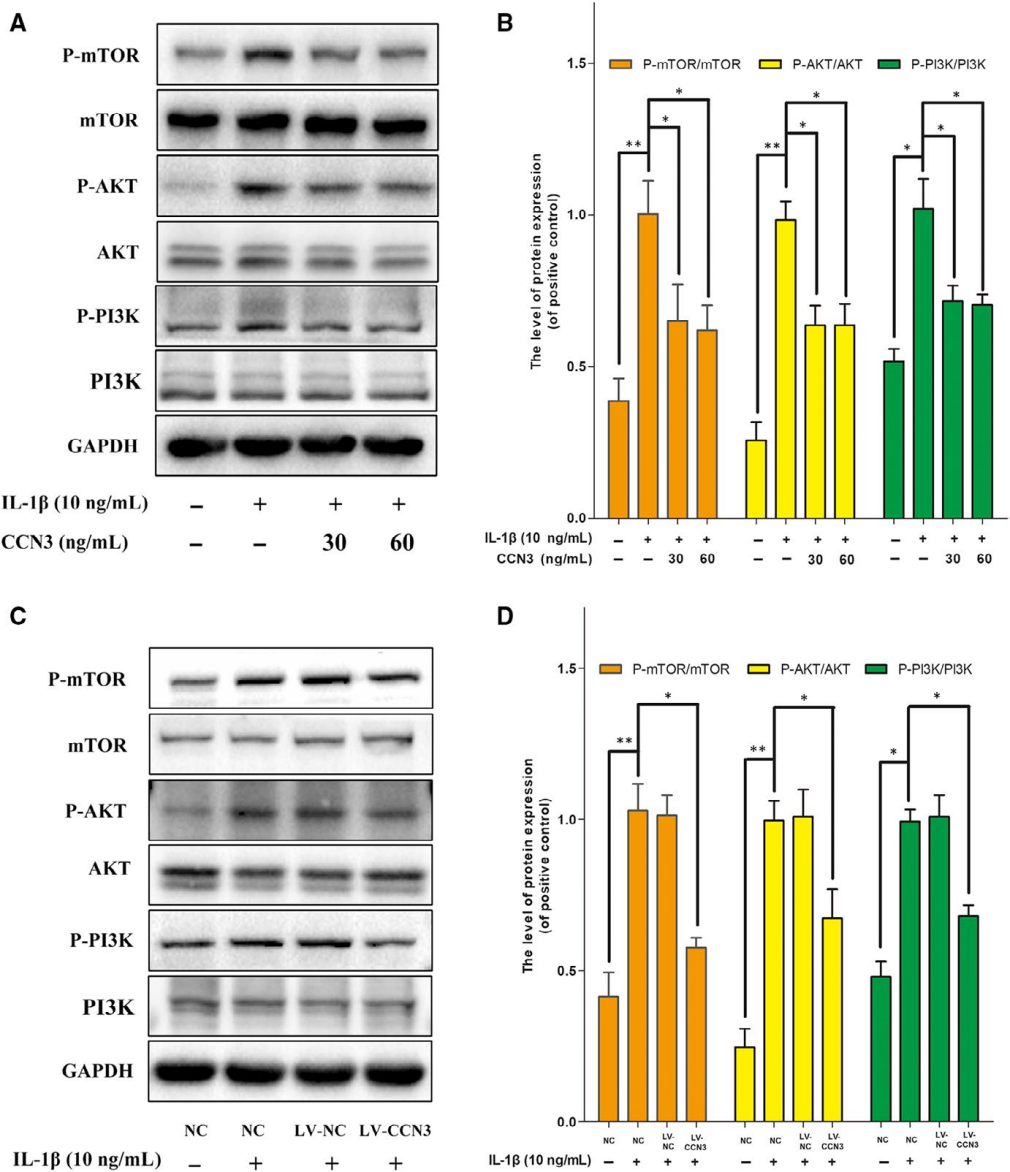
CCN3 induces cartilage protection via blocking the PI3K/AKT/mTOR pathway. A, SW1353 cells were treated with recombinant CCN3 (30, 60 ng/mL) and IL‐1β (10 ng/mL) for 30 min. Western blot showed recombinant CCN3 attenuated the increased protein levels of P‐PI3K, P‐AKT and P‐mTOR activated by IL‐1β. B, Quantification of P‐PI3K, P‐AKT and P‐mTOR immunoblots. C, CCN3 overexpression cells were treated IL‐1β (10 ng/mL) for 30 min. Western blot showed CCN3 overexpression attenuated the increased protein levels of P‐PI3K, P‐AKT and P‐mTOR activated by IL‐1β. D, Quantification of P‐PI3K, P‐AKT and P‐mTOR immunoblots. The data represent the mean ± SD of three independent experiments, each performed in triplicate. Significant differences between groups are indicated as ***P < .001, **P < .01 and *P < .05

The PI3K/AKT/mTOR signalling pathway is central fora plethora of cellular mechanisms. In our experiment, we examined the effects of recombinant CCN3 (30, 60 ng/mL) on the activation of the PI3K/AKT/mTOR pathway. As shown in Figure 7A,B, CCN3 attenuated the increased protein levels of P‐PI3K, P‐AKT and P‐mTOR activated by IL‐1β. Moreover, we observed that CCN3 overexpression inhibited the activation of the PI3K/AKT/mTOR pathway (Figure 7C,D).

## 4 DISCUSSION

In this study, we confirmed that CCN3 expressions was decreased in human and rat knee OA articular cartilage. Recombinant CCN3 ameliorated the IL‐1β‐induced matrix catabolism, as demonstrated by MMP1, MMP3, MMP13, ADAMTS5 and iNOS expression, in vitro. In addition, the degradation of cartilage matrix such as collagen 2 and aggrecan could be reversed by CCN3. Furthermore, we found that CCN3 promoted autophagy as Atg5, Beclin1 and LC3‐II expression were increased. HMGB1 was negatively correlated with CCN3 in osteoarthritis, and HMGB1 is involved in the protective effect of CCN3 in OA. Moreover, CCN3 overexpression decreased the expression of HMGB1 and reversed the IL‐1β induced MMP production. Additionally, recombinant CCN3 or CCN3 overexpression attenuated the PI3K/AKT/mTOR pathway activation.

CCN3 is a member of the CCN (Cyr61, Ctgf, NOV) family and is capable of regulation inflammation, wound healing, osteogenesis and cell differentiation.^21^ CCN3 exerted anti‐inflammatory effects in endothelial cells via the inhibition of NF‐κB nuclear accumulation.^22^ CCN3 overexpression attenuated the inflammatory progress of atherosclerosis in apolipoprotein E‐deficient mice.^23^ In addition, a previous study reported that CCN3 attenuated inflammatory pain through regulation of MMP2 and MMP9, indicating that CCN3 could function as a modulator of inflammatory mediators.^24^ Previous investigations reported that mice lacking the CCN3 protein might cause OA‐like disease, but the underlying mechanism remained unclear. In our study, western blot and immunohistochemical analysis of CCN3 in human and rat OA cartilage tissue confirmed that CCN3 expression was decreased in OA, which was consistent with previous studies.^19^, ^25^

The loss of articular cartilage is a prominent sign of OA. It is commonly accepted that the overproduction of MMPs and ADAMTs causes cartilage matrix degradation.^26^ Autophagy is an intracellular reaction that degrades proteins and organelles to maintain cellular homeostasis,^27^ and it is essential for maintaining the integrity and function of articular cartilage.^28^ Mice lacking the autophagy regulator Atg5 display chondrocyte apoptosis and histopathological signs of knee joint OA.^29^ IL‐1β plays a crucial role in the pathogenesis of OA. It induces inflammatory reactions and degeneration articular cartilage matrix degeneration.^30^ In this study, SW1353 cell were treated with IL‐1β to simulate OA‐like change in vitro. Our data showed that recombinant CCN3 ameliorated the IL‐1β‐induced catabolism, cartilage matrix degradation and promoted autophagy in vitro. These results indicated that CCN3 exerted anti‐inflammation properties and could promote autophagy during OA.

High‐mobility group box 1 is the best characterized damage‐associated molecular pattern (DAMP) molecules.^31^ Upon cell activation or injury, HMGB1 is transported from the nucleus to the cytoplasm. Extracellular HMGB1 is involved in inflammasome activation as well as in the regulation of the autophagy/apoptosis balance.^32^ Previous studies have demonstrated that HMGB1 acts as a key pro‐inflammatory cytokine in various inflammatory disorders.^33^ High‐mobility group box 1 is overproduced and relocated in synovial membranes of patients with knee OA. Besides, HMGB1 levels in synovial fluid reflects the severity of OA.^34^ High‐mobility group box 1 can synergize with IL‐1β to amplify the inflammatory response, resulting in the production of a large number of cytokines, chemokines, and MMPs.^10^ We thus suspect that CCN3 may protect against osteoarthritis via HMGB1 modulation. Our data showed that HMGB1 was negatively correlated with CCN3 expression in IL‐1β‐induced osteoarthritis responses. Recombinant CCN3 or CCN3 overexpression reduced HMGB1 expression and reversed the IL‐1β induced MMP and HMGB1 production. These findings indicate that HMGB1 is involved in the protective effect of CCN3 in OA. However, the detailed regulatory relationship between CCN3 and HMGB1 remains further exploration.

The PI3K/AKT/mTOR signalling pathway plays a critical role in regulating diverse cellular processes including cell proliferation, apoptosis, metabolism, differentiation and cell cycle.^35^ mTOR is a major repressor of autophagy and is a downstream target of the PI3K and AKT pathway.^36^ Previous studies have showed that both pharmacological inhibition and genetic deletion of mTOR can increase the severity of OA in preclinical mouse models.^37^ Moreover, blockade of PI3K/AKT/mTOR signalling pathway could promote the autophagy of articular chondrocytes and attenuate inflammation response in rat chondrocytes.^38^ The results of the present study indicated that recombinant CCN3 (30, 60 ng/mL) decreased the levels of phosphorylated PI3K, AKT and mTOR activated by IL‐1β. Besides, the activated PI3K/AKT/mTOR pathway could also be ameliorated by CCN3 overexpression. These results indicated that the PI3K/AKT/mTOR pathway might be involved in the protective effects of CCN3 against OA.

To summarize, we have shown that CCN3 ameliorates the IL‐1β‐induced catabolism, cartilage matrix degradation and promotes autophagy process by suppressing PI3K/AKT/mTOR signalling pathway. High‐mobility group box 1 is involved in the protective effect of CCN3 in OA. This study explored the new protective mechanism of CCN3 in osteoarthritis and noted that CCN3 could serve as a novel potential therapeutic target for osteoarthritis.

## CONFLICT OF INTEREST

The authors confirm that there is no conflict of interest.

## AUTHORS' CONTRIBUTIONS

Xiaojian Huang and Hongbo You designed the study; Xiaojian Huang, Bowei Ni and Yang Xi performed the experiments; Zhekai Mao, Xiangyu Chu, Rui Zhang and Xiaohu Ma analysed the results and approved the publication; Xiaojian Huang prepared the first draft; Hongbo You revised the paper.

## Data Availability

The data that support the findings of this study are available from the corresponding author upon reasonable request.
